# Untargeted metabolomic profiling in a patient with glycogen storage disease Ib receiving empagliflozin treatment

**DOI:** 10.1002/jmd2.12304

**Published:** 2022-05-22

**Authors:** Eran Tallis, Cecile L. Karsenty, Amanda B. Grimes, Lina B. Karam, Sarah H. Elsea, Vernon Reed Sutton, Brandy L. Rawls‐Castillo, Ning Liu, Claudia Soler‐Alfonso

**Affiliations:** ^1^ Department of Molecular and Human Genetics Baylor College of Medicine Houston Texas USA; ^2^ Department of Pediatrics Baylor College of Medicine Houston Texas USA; ^3^ Texas Children's Cancer and Hematology Centers Houston Texas USA; ^4^ Department of Pediatrics‐Gastroenterology Baylor College of Medicine Houston Texas USA; ^5^ Baylor Genetics Houston Texas USA

**Keywords:** global metabolomics, hypoglycemia, inborn errors of metabolism, inflammatory bowel disease, neutropenia

## Abstract

Glycogen storage disease type Ib (GSD‐Ib) is a rare inborn error of glycogen metabolism uniquely associated with neutropenia and neutrophil dysfunction, causing severe infections, inflammatory bowel disease (IBD), and impaired wound healing. Recently, kidney sodium‐glucose co‐transporter‐2 (SGLT2) inhibitors such as empagliflozin known to reduce plasma levels of 1,5‐anhydroglucitol (1,5‐AG) and its toxic derivatives in neutrophils, have been described as a new treatment option in case reports of patients with GSD‐Ib from Europe and Asia. We report our experience with an 11‐year‐old girl with GSD‐Ib presenting with short fasting hypoglycemia, neutropenia with neutrophil dysfunction, recurrent infections, suboptimal growth, iron‐deficiency anemia, and IBD. Treatment with daily empagliflozin improved neutrophil counts and function with a significant reduction in G‐CSF needs. Significant improvement in IBD has led to weight gain with improved nutritional markers and improved fasting tolerance. Reduction of maximum empagliflozin dose was needed due to arthralgia. No other significant side effects of empagliflozin were observed. This report uniquely highlights the novel use of untargeted metabolomics profiling for monitoring plasma levels of 1,5‐AG to assess empagliflozin dose responsiveness and guide dietary management and G‐CSF therapy. Clinical improvement correlated to rapid normalization of 1,5‐AG levels in plasma sustained after dose reduction. In conclusion, empagliflozin appeared to be a safe treatment option for GSD‐Ib‐associated neutropenia and neutrophil dysfunction. Global untargeted metabolomics is an efficient method to assess biochemical responsiveness to treatment.

## 1 INTRODUCTION

Glycogen storage disease type Ib (GSD‐Ib, OMIM# 232220) is caused by pathogenic variants in the *SLC37A4* gene, which encodes the glucose‐6‐phosphate transporter (G6PT). G6PT is localized in the endoplasmic reticulum (ER)membrane, where it facilitates the translocation of glucose‐6‐phosphate (G6P) from the cytoplasm into the ER lumen and its hydrolysis into glucose and phosphate.^1^ GSD‐Ib is clinically characterized by severe fasting hypoglycemia, hepatomegaly, truncal obesity, short stature, hyperuricemia, hyperlipidemia, and elevated lactate levels. Additionally, patients with GSD‐Ib develop neutropenia and inflammatory bowel disease (IBD),^2^, ^3^ presenting with recurrent infections, chronic diarrhea, abdominal pain, growth failure, anemia, and mucosal ulcerations. Neutropenia is caused by decreased production of mature neutrophils and enhanced apoptosis.^3^, ^4^, ^5^ Neutrophil dysfunction is reflected by impaired respiratory burst, defective chemotaxis, and abnormal calcium flux.^5^, ^6^, ^7^ G6PT‐deficient neutrophils show reduced glucose utilization characterized by decreased glucose uptake and reduced levels of intracellular G6P, lactate, ATP, and NADP,^5^ with subsequent impairment in adhesion and migration.^8^

G6PT transports not only G6P, but also its structural analog 1,5‐anhydroglucitol‐6‐phosphate (1,5‐AG‐6P). Veiga‐da‐Cunha et al.^9^ have recently shown that the impaired glucose utilization of G6PT‐deficient neutrophils is caused by failure to eliminate 1,5‐AG‐6P. Accumulation of 1,5‐AG‐6P strongly inhibits hexokinases that catalyze the first step of glycolysis, resulting in energy deficiency in neutrophils and subsequent apoptosis. 1,5‐AG‐6P is made by enzymatic side reactions from 1,5‐anhydroglucitol (1,5‐AG), a non‐degradable glucose analog present in blood.^10^

Studies in a G6PC3‐deficient mouse model that phenotypically and biochemically mimics neutrophil impairment of patients with GSD‐Ib have shown that treatment with an inhibitor of the kidney sodium glucose co‐transporter 2 (SGLT2) was able to lower blood levels of 1,5‐AG‐6P and consequently restore a normal neutrophil count.^9^ SGLT2 inhibitors, such as empagliflozin, are anti‐diabetic medications that inhibit renal glucose reabsorption.^11^ Glucosuria decreases renal 1,5‐AG reabsorption and thereby lowers its plasma concentrations, subsequently reducing the concentration of its toxic derivative 1,5‐AG‐6P in neutrophils.^12^, ^13^ First clinical reports from Europe and Japan with seven GSD‐Ib patients receiving empagliflozin showed very encouraging results with improvement in neutrophil count and function.^12^, ^13^, ^14^, ^15^ We hereby describe our experience with empagliflozin treatment in a patient with GSD‐Ib, highlighting a novel use of global untargeted metabolomics to ascertain normalization of 1,5‐AG‐6P by measuring 1,5‐AG in plasma.

## 2 CASE REPORT

The patient is an 11‐year‐old girl, who was diagnosed with GSD‐Ib at the age of 4 months following evaluation for hypoglycemia, hyperlipidemia, and hepatomegaly. Liver biopsy demonstrated accumulation of glycogen in hepatocytes consistent with GSD. Molecular testing found compound heterozygous variants in the *SLC37A4* gene (c.1042_1043del (p.Leu348fs), classified as pathogenic, and IVS1+1G>T, classified as a variant of unknown significance), ascertaining the diagnosis. The patient was started on a standard GSD diet with fructose and galactose restriction, as well as frequent cornstarch doses. Despite dietary management, she continued to show severe episodes of hypoglycemia, requiring frequent feeds every 2 h to maintain normal glucose levels. Despite dietary management, she continued to show severe episodes of hypoglycemia over the years, leading to nine hypoglycemic seizures.

**FIGURE 1:**
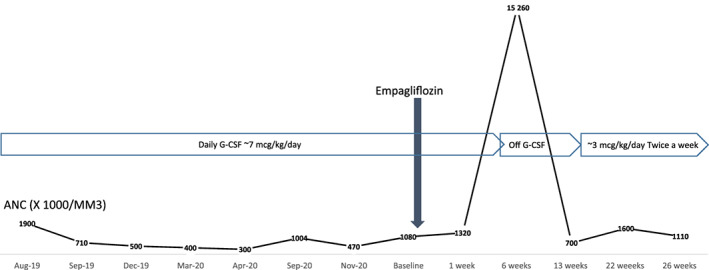
Absolute neutrophil count (ANC) response to empagliflozin therapy

At the age of 7 months, the patient was found to be profoundly neutropenic with an absolute neutrophil count (ANC) of 7210^3^/μl (normal: 1.63–7.87 × 10^3^/μl). Daily granulocyte colony‐stimulating factor (G‐CSF) therapy at an average dose of 3 mcg/kg/day was needed to maintain ANC levels between 300‐800 x 10^3^/μl over the subsequent ~10 years. In addition to quantitative neutropenia, neutrophil oxidative burst assay showed severe neutrophil dysfunction, with 0% neutrophil activation. She experienced several episodes of opportunistic infections, including recurrent episodes of skin and soft tissue infections with at least five episodes requiring intravenous antibiotics and drainage of abscesses (Figure 1).

At 2 years of age, she was found to have suboptimal growth, iron‐deficiency anemia, elevated inflammatory markers, recurrent abdominal pain, and diarrhea. She was diagnosed with GSD‐related IBD, and started treatment with sulfasalazine followed by mesalamine due to severe IBD exacerbations.

## 3 METHODS

Consent for off‐label use of empagliflozin was obtained from parents after a detailed explanation regarding treatment rationale, prior published case reports, possible side effects, and surveillance regimen. Subsequent consent for the publication of anonymized clinical data was also obtained from legal guardians. Empagliflozin was started at 0.48 mg/kg/day divided every 12 h, well within the dosage window of 0.3–0.7 mg/kg/day used in prior reports.^12^, ^13^, ^14^, ^15^ After 2 weeks without reported side effects from treatment, the dose was increased to 0.6 mg/kg/day. Post‐intervention evaluations were done at 1, 6, 13, and 22 weeks of treatment. The patient was monitored closely by finger‐stick blood glucose measurements and a continuous glucose monitor. Frequent dietary assessments by a metabolic dietitian were completed. Data regarding G‐CSF requirements, mouth sores, and infections were collected. Laboratory evaluation included complete blood counts with ANC, complete metabolic panel including liver function tests, lipid profile, prealbumin, uric acid, erythrocyte sedimentation rate (ESR), C‐reactive protein (CRP), and fecal calprotectin. Neutrophil oxidative burst studies were completed by measuring NADPH oxidase activity using dihydrorhodamine fluorescence in different populations of immature and mature neutrophils. Measurement of 1,5‐AG in plasma was obtained by global untargeted metabolomics analysis. Global untargeted metabolomics profiling (global MAPS®) uses systematic identification and quantitation of metabolites in biological samples by using ultra‐performance liquid chromatography coupled with high‐resolution mass spectrometry as described previously.^16^, ^17^ Small molecules ranging in size from 75 to 1000 Da in the biofluids are identified. Z‐scores are calculated as the standard deviations from the mean of a normal reference cohort consisting of ~400 healthy individuals.^16^ To assess IBD response, the patient underwent magnetic resonance enterography at 15 weeks of treatment, as well as frequent assessments using the pediatric Crohn's disease activity index (PCDAI), a validated instrument for measuring disease activity in children and adolescents with Crohn's disease.^18^, ^19^ Disease activity is based on evaluating abdominal pain, the number of daily stools, hematocrit, ESR, and albumin levels. Scores range from 0 to >40, representing remission, mild, moderate, or severe disease.^18^, ^19^

## 4 RESULTS

Empagliflozin treatment was started at 10 mg every 12 h by enteral administration (0.48 mg/kg/day). The patient tolerated this dose well with no significant adverse events. After 10 days of treatment, the dose was increased to 25 mg daily (0.6 mg/kg/day). She initially tolerated the dose without adverse events. However, 2 weeks after the dose increase, she reported significant daily generalized arthralgia.

Consequently, empagliflozin dose was decreased to the initial dose of 10 mg every 12 h with complete resolution of the arthralgia within 1 week. No other side effects were noted. Importantly, there have been no significant episodes of hypoglycemia attributed to treatment.

Shortly after starting empagliflozin treatment, significant improvement was observed in clinical and laboratory assessments (Table 1). Untargeted metabolomics showed immediate improvement in 1,5‐AG levels in plasma, which was significantly elevated before treatment (Z score of +3.2; normal: −2.0 to +2.0). It quickly decreased to normal range after 1 week of treatment (Z score of −0.15). 1,5‐AG has remained normal since. Untargeted metabolomic profiling also detected elevated GSD markers, such as urate and pentose metabolism intermediates (pseudouridine, orotidine), with normalization of these metabolites following therapy. Metabolomic profiling also detected low Z‐scores for fructose, high Z‐scores for branched‐chain amino acids, and elevations of various plasmalogens, phosphatidylethanolamines, and phosphatidylcholine when compared to the mean normal reference with no change after initiation of empagliflozin treatment.

**TABLE 1:** Growth parameters and chemistry values in a GSD Ib patient using empagliflozin

Weeks of treatment	Weight (kg)	Height (cm)	BMI (kg/m^2^)	IBD score (PCDAI^a^)	CRP (<1 mg/dl)	Prealbumin (18–44 mg/dl)	Uric acid (2–6.2 mg/dl)	Lactate (0.2–2 mmol/L)	1,5‐Anhydroglucitol Z score (−2 to +2)	Fasting intervals (average)
Baseline	41.4	135.3	22.62	Moderate	14.8	11.7	7.2		Elevated (+3.2)	Every 2 h
1 week	41.4	135.3	22.62	Mild	1	22.5	6.9	0.8	Normal (−0.15)	Every 2 h
6 weeks	43.9	136.4	23.6	Remission	0.5	23.3	6.8	3.6	Low (−2.1)	Every 3 h
13 weeks	46.9	136	25.36	Remission	<0.5	25	5.5	1.7	Normal (−1.67)	Every 3–4 h
22 weeks	47.9	137	25.57	Remission	<0.5	24.5	5.8	1.6	Low‐normal (−1.96)	Every 3 h

^a^ PCDI: pediatric Crohn's disease activity index.

**FIGURE 2:**
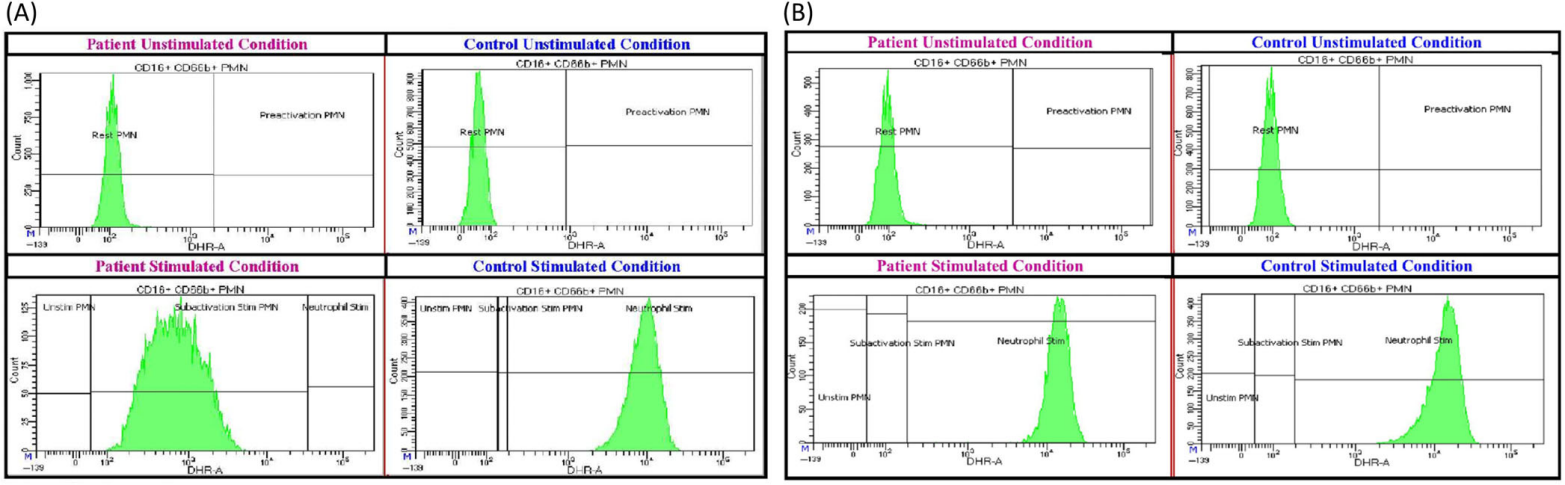
Neutrophil function test before and following empagliflozin therapy

Following empagliflozin treatment, ANC has markedly improved, fluctuating between 700 and 15 960 x 10^3^/μl (normal: 1.63–7.87 × 10^3^/μl) (Figure 1 and 2A). Neutrophil function improved significantly with near normalization of neutrophil oxidative burst after 1 week of empagliflozin therapy and complete normalization after 6 weeks (Figure 2B). She has not had further episodes of infections and has demonstrated near‐complete resolution of episodic oral ulcers. Following improvement in ANC and neutrophil function, G‐CSF has slowly been weaned from daily dosing to twice weekly dosing, at ~3 mcg/kg/dose. Specifically, the patient continued daily G‐CSF dosing throughout the first 6 weeks of therapy. However, following markedly elevated ANC (to 15 260/μl), G‐CSF was held altogether, with the patient requiring only infrequent, intermittent doses over the next 4 weeks. She subsequently developed an oral ulcer, with ANC dropping to 700–800/μl. G‐CSF therapy was resumed to every other day, following 10 weeks of empagliflozin therapy. G‐CSF frequency was weaned further to every 72 h after 16 weeks of empagliflozin therapy, then to twice weekly dosing after 20 weeks of empagliflozin therapy (still at 3 mcg/kg/dose). In the subsequent weeks, on continued empagliflozin therapy, the patient developed only intermittent, less severe oral ulcers, with ANC trough values between 1000/μl and 2000/μl.

Along with the improved neutrophil count and function, abdominal pain, diarrhea, and overall well‐being improved quickly as reflected by the PCDAI, which dropped from 35 (moderate activity) before treatment to 27.5 (mild) after only 1 week of treatment, and to 5 (remission) within 6 weeks of treatment, with resolution of IBD symptoms. Inflammatory markers significantly improved; CRP dropped from 14.8 to 1 mg/dl (normal: <1.0 mg/dl) after the first week of treatment, and to 0.5 mg/dl at 6 weeks of treatment. MR enterography performed 15 weeks after the start of treatment did not show any imaging signs of small bowel or colonic inflammation. With IBD response, patient's weight increased from 41.4 kg (71st percentile) to 46.9 kg (84th percentile). Other nutritional markers showed improvement including prealbumin increase from 11.7 to 25 mg/dl (normal: 18–44 mg/dl), and total cholesterol increase from 93 to 150 mg/dl (normal: 112–208 mg/dl). Uric acid, a GSD specific marker, improved from 7.2 to 5.5 mg/dl (normal: 2.0–6.2 mg/dl).

Dietary management upon starting empagliflozin treatment included meals every 2 h with galactose and fructose restriction to decrease sources of gluconeogenesis and excessive production of G6P. She consumed roughly 75% carbohydrates, 12% protein and 13% fat in her diet. Her daily caloric intake averaged 38 kcal/kg/day (daily recommended allowance for age and gender 42 kcal/kg/day). She received uncooked cornstarch (UCS) every 2–3 h at an average of 0.5–1 g/kg/dose (ACMG guidelines^20^ recommendations 1.6–2.5 g/kg/day). Twelve weeks into treatment, she was able to tolerate spacing meals and UCS doses to every 3 h. Her appetite has also increased significantly. She was consuming 60% carbohydrates, 21% protein, and 19% fat in her diet. Her overall caloric intake increased from 1592 calories per day to an average of 2390 calories per day. Attempts to space fasting intervals to every 4 h resulted in episodes of hypoglycemia. Due to improved weight and increase in fasting intervals and IBD symptoms, all protein supplementation was weaned off her diet.

## 5 DISCUSSION

Accumulation of 1,5‐AG‐6P in neutrophils leads to neutrophil dysfunction and neutropenia in individuals with GSD‐Ib. Uncovering this mechanism has led to off‐label treatment trials with empagliflozin. Empagliflozin is registered for treating type 2 diabetes in adults and has a favorable safety profile.^21^ The most described adverse effects are increased thirst and urogenital fungal skin infections resulting from glucosuria. Other side effects include urinary tract infections, hyperlipidemia, and arthralgias. Hypoglycemia was described only when empagliflozin was combined with insulin or insulin secretagogues (e.g., sulfonylurea) in diabetic patients. Our patient tolerated the initial dose (0.48 mg/kg/day) well with no severe side effects. She developed generalized arthralgia shortly after increasing her dose to 0.6 mg/kg/day, despite previous case reports using up to 0.7 mg/kg/day without side effects. The treatment dose was decreased with complete resolution of arthralgias. No hypoglycemic episodes directly related to treatment occurred in the patient, even on the higher dose.

Consistent with previous reports,^12^, ^13^, ^14^, ^15^ our patient showed rapid and significant improvement in all clinical and laboratory parameters that started within the first 7 days of treatment. Initiation of empagliflozin treatment enabled weaning of G‐CSF, thereby minimizing risks for myelodysplasia, and acute myeloid leukemia, associated with prolonged G‐CSF use.^22^, ^23^ Reduced bowel inflammation led to improved enteral absorption that allowed spacing of her feeds from every 2 h to every 3 h. This has never been possible before empagliflozin treatment and has been very impactful for the patient's and family's quality of life.

Improvement correlated to normalization of 1,5‐AG levels in plasma. 1,5‐AG is the 1‐deoxy form of glucose. 1,5‐AG is found in high quantities in soybeans, rice, bread, beef, and other plant sources. 1,5‐AG is freely filtered by glomeruli and is almost entirely reabsorbed by the proximal renal tubule. In hyperglycemia, the excess glucose competes with 1,5‐AG for reabsorption, which in turn increases urine excretion of 1,5‐AG.^24^, ^25^ Thus, 1,5‐AG has been previously studied as a biomarker of metabolic control in patients with diabetes. Quantification of 1,5‐AG is available as a registered clinical test (GlycoMark®), validated for assessment of glycemic control only in patients with diabetes mellitus type II.^25^ Novel use of untargeted metabolomic profiling with quantification of 1,5‐AG in our patient detected significantly increased 1,5‐AG before treatment, with subsequent rapid normalization following treatment, correlating to improvement in neutrophil numbers and function. 1,5‐AG remained within normal limits even with increased appetite, consumption of different foods, and increased fasting tolerance. Untargeted metabolomics also showed improvement with treatment in other parameters of metabolic control in GSD patients, including urate and urate intermediates. GSD dietary management was reflected with persistently low fructose and high branched‐chain amino acid intermediates secondary to strict fructose avoidance and protein supplementation in this patient. Lastly, alterations in other metabolic pathways, most noticeably fatty acids and lipids metabolism were observed using metabolomic profiling, similarly to a previous report by Mathis et al.^26^ Our data suggest that untargeted metabolomics is a useful tool for assessment of metabolic control and adherence to dietary management in patients with GSD using empagliflozin. Likewise, quantification of 1,5‐AG using untargeted metabolomic profiling may be used as a biomarker for future clinical trials of empagliflozin in the GSD‐Ib population.

In summary, our data show that empagliflozin is a safe and promising new option for the treatment of patients with GSD‐Ib, IBD, and neutropenia. In our patient, empagliflozin seems to be superior to G‐CSF with respect to correction of neutrophil dysfunction and side effects. Global untargeted metabolomics appears to be an efficient method to assess biochemical responsiveness to treatment. Results from a formal clinical trial are needed to established safety, tolerability, and efficacy of empagliflozin in patients with GSD‐Ib.

## AUTHOR CONTRIBUTIONS

**Eran Tallis**: planned, conducted, and reported the work described in the article. **Cecile L. Karsenty**: conducted and reported on the work described in the article. **Amanda B. Grimes**: conducted and reported on the work described in the article. **Lina B. Karam**: conducted and reported on the work described in the article. **Sarah H. Elsea**: conducted and reported on the work described in the article. **V. Reed Sutton**: conducted and reported on the work described in the article. **Brandy L. Rawls‐Castillo**: conducted and reported on the work described in the article. **Ning Liu**: conducted and reported on the work described in the article. **Claudia Soler‐Alfonso**: planned, conducted, and reported the work described in the article.

## FUNDING INFORMATION

None.

## CONFLICTS OF INTEREST

Eran Tallis declares that he has no conflict of interest. Cecile L. Karsenty declares that he has no conflict of interest. Amanda B. Grimes declares that he has no conflict of interest. Lina B. Karam declares that he has no conflict of interest. Sarah H. Elsea declares that he has no conflict of interest. V. Reed Sutton declares that he has no conflict of interest. Brandy L. Rawls‐Castillo declares that he has no conflict of interest. Ning Liu declares that he has no conflict of interest. Claudia Soler‐Alfonso declares that he has no conflict of interest.

## INFORMED CONSENT

Informed consent was obtained from all patients for being included in the study. Proof that informed consent was obtained IS available upon request.

## CONSENT FOR IDENTIFIABLE INFORMATION

No identifiable information is used in this report.

## ANIMAL RIGHTS

This article does not contain any studies with human or animal subjects performed by any of the authors.

## ETHICS STATEMENT

All procedures followed were in accordance with the ethical standards of the responsible committee on human experimentation (institutional and national) and with the Helsinki Declaration of 1975, as revised in 2000 (5).

## Data Availability

The authors confirm that the data supporting the findings of this study are available within the article. Supplementary materials are not available. The manuscript has no associated data.
